# TRPA1 mediated aggravation of allergic contact dermatitis induced by DINP and regulated by NF-κB activation

**DOI:** 10.1038/srep43586

**Published:** 2017-02-27

**Authors:** Jun Kang, Yong Ding, Baizhan Li, Hong Liu, Xu Yang, Mingqing Chen

**Affiliations:** 1 Hubei Key Laboratory of Genetic Regulation and Integrative Biology, School of Life Sciences, Central China Normal University, Wuhan 430079, Hubei, China; 2 Key Laboratory of the Three Gorges Reservoir Region’s Eco-Environment, Ministry of Education, Chongqing University, Chongqing 400045, China

## Abstract

The possible pathogenic role and mechanism of Di-iso-nonyl phthalate (DINP) in allergic dermatitis is still controversial. This work has shown that oral exposure to DINP exacerbated allergic dermatitis tissue lesions in FITC-sensitized mice. The lesions was accompanied by an enhancement of TRPA1 expression and an increase in IgG1, IL-6 and IL-13 levels. This work also found that blocking TRPA1 by HC030031 effectively prevented the development of allergic dermatitis resulting from oral exposure to DINP and/or FITC-sensitized mice. This result is marked by the down regulation of IgG1 levels, a reduction in mast cell degranulation and a decrease in IL-6 and IL-13 levels. We also showed that blocking NF-κB inhibited TRPA1 expression, and that blocking TRPA1 had no significant effect on the activation of NF-κB or TSLP expression. This study helps in understanding the role DINP exposure plays in the development of allergic dermatitis and provides new insight into the mechanisms behind the DINP-induced adjuvant effect.

Phthalates are widely used as plasticizers and can be found in PVC, toys, food packaging materials, medical bags and hoses, vinyl flooring, personal care products and hundreds of other products^1^,^2^,^3^. Di-iso-nonyl phthalate (DINP) is now the most widely used plasticizer due to its low toxicity^4^,^5^. The major routes of DINP exposure are through diet and the use of personal care products. Several studies have documented an association between phthalate exposure and an increased risk of allergies^6^,^7^,^8^. DINP and di-iso-decyl phthalate metabolites were reported to increase the risk of asthma in Norwegian children^9^. Animal data suggests that DINP may worsen dermatitis and upregulated interleukin-4 levels^6^,^10^. However, the association between DINP exposure and an increased risk of allergic dermatitis is still controversial. Several studies have indicated that some phthalates may increase allergic responses by acting as adjuvants for the production of cytokines and/or immunoglobulins that are responsible for allergic sensitization^6^,^7^,^8^. The possible pathogenic role and mechanisms of DINP in allergic dermatitis need to be clarified.

Allergic contact dermatitis (ACD) is a delayed-type hypersensitivity response, which is characterized by contact with allergens, causing tissue inflammation^11^, and accompanying itching^12^,^13^,^14^. Inflammation leads to eczema and skin barrier breakdown^14^. The main feature of ACD is persistent itching, and scratching behavior can lead to further damage which promotes allergen and pathogen invasion of the skin^15^.

An itchy feeling is started by sensory nerve fibers innervating the dermal inflammation site^16^,^17^. It has been shown that contact hypersensitivity (CHS) is eliminated through removal of capsaicin-sensitive nerve fibers^18^,^19^. Transient receptor potential (TRP) ion channels are suggested to mediate acute inflammatory and pruritic responses following exogenous stimulation, and may contribute to allergic responses^20^. Histamine receptors have been shown to stimulate sensory nerve fibers by activating a TRP ion channel subfamily V, member, 1 (TRPV1) in rodent models^20^,^21^. Another member of a TRP cation channel, subfamily A, member 1 (TRPA1), plays a dual role in conducting pain and itchiness^20^. This channel proved to be essential for pruritus induced in mice by injection of chloroquine^22^. A recent study reported that TRPA1 antagonist therapy can rebuild the skin barrier and reduce scratching behavior in an ACD mouse model^23^. TRPA1 agonist has been shown to enhance skin sensitization by an FITC sensitized mouse model^24^.

The cytokine Thymic stromal lymphopoietin (TSLP) expressed mainly by epithelial cells, has been recognized as a key player in the pathogenesis of allergy diseases^25^. It primes and stimulates DC maturation, and enhances the recruitment of Th2 effector cells^26^. TSLP gene expression is regulated by transcription factor NF-κB^27^. Our recent study suggested that DINP exacerbated oxidative stress and activation of NF-κB^28^. However, little is known about the relationship between a TRPA1 channel and the activation of NF-κB in CHS.

In this paper, we first investigated whether a DINP exposure-induced CHS response involved a TRPA1 channel. We then attempted to determine whether activation of a TRPA1 channel is dependent on the activation of NF-κB in an ACD mouse model.

## Results

### DINP exacerbated the expression of TRPA1 in an FITC-induced ACD mouse model

**Figure 1:**
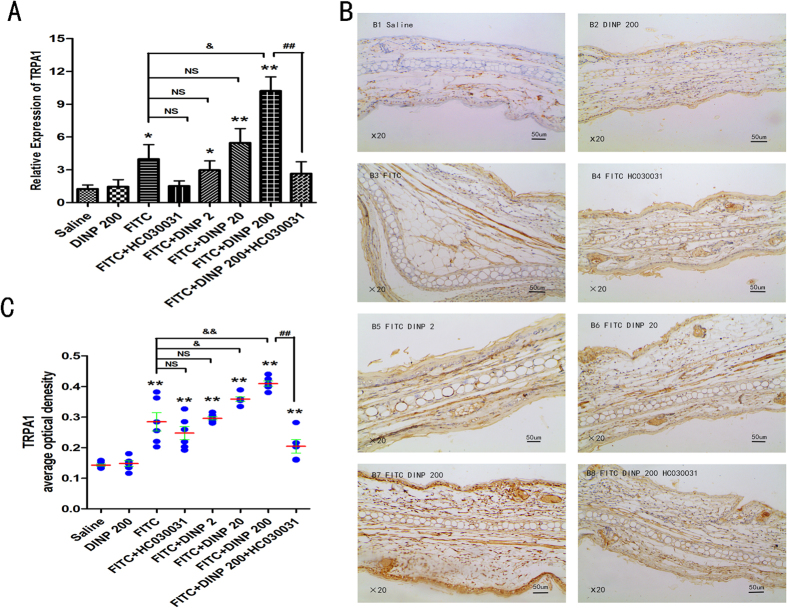
DINP exacerbated expression of TRPA1 and HC030031 inhibited this effect. (**A**) The expression levels of TRPA1 mRNA. (**B**) Immunohistochemistry for TRPA1. (**C**) The expression scores of TRPA1. *p < 0.05, **p < 0.01, compared with saline group. NS, no significant difference, ^&^p < 0.05, ^&&^p < 0.01 compared with FITC group. ^##^p < 0.01, FITC + DINP200 group versus FITC + DINP200 + HC030031 group. (n = 7). Saline group, mice were gavaged with saline for 3 weeks, then sensitized and challenged with saline and vehicle; DINP200 group, mice were gavaged with 200 mg/(kg·d) of DINP for 3 weeks, then sensitized and challenged with saline and vehicle saline; FITC group, mice were gavaged with saline for 3 weeks, then sensitized and challenged with 0.5% FITC; FITC + HC030031 group, mice were gavaged with saline for 3 weeks, on days 22, 23 and 28, HC030031 was injected (50 mg/kg, i.p.) before sensitization and challenge with 0.5% FITC; FITC + DINP2, FITC + DINP20, FITC + DINP200 groups, mice were gavaged with 2, 20 and 200 mg/(kg·d) dose of DINP for 3 weeks, then sensitized and challenged with 0.5% FITC; FITC + DINP200 + HC030031 group, mice were gavaged with 200 mg/(kg·d) dose of DINP for 3 weeks, on days 22, 23 and 28, HC030031 was injected (50 mg/kg, i.p.) before sensitization and challenge with 0.5% FITC.

To investigate the molecular mechanisms behind DINP exacerbated allergic dermatitis, we investigated the expression of TRPA1 in the skin of ears after the mice were exposed to DINP by oral gavage and sensitized with FITC. It was found that FITC induced the upregulation of TRPA1 mRNA levels in the skin of ears, comparing the four FITC exposure groups with the saline-only group (Fig. 1A). It should be pointed out that exposure to DINP alone did not result in an increase in TRPA1 expression, as seen by comparing the DINP-200 exposure mice with the saline-only group. Importantly, compared to the groups exposed only to FITC, the expression of TRPA1 was sharply enhanced in the groups treated with DINP and FITC. TRPA1 expression increased with increasing of DINP exposure (Fig. 1A).

The changes of TRPA1 expression upon DINP and FITC were further examined by immunohistochemical analysis (Fig. 1B,C). A marked increase in TRPA1 expression in response to DINP exposure and FITC treatment has been validated, although exposure to DINP alone did not result in a significant increase in TRPA1 expression (Fig. 1B,C). Interestingly, the changes in TRPA1 expression were consistent with the effect of allergic contact dermatitis responding to DINP and FITC described in our previous work^28^.

HC030031 was used to block activation of the TRPA1 ion channel. After treatment with HC030031, we examined the expression of TRPA1 in the mouse ear by using real time RT-PCR and immunohistochemical analysis. It was found that TRPA1 expression was inhibited by HC030031, which was demonstrated by comparing the FITC + HC030031 group to the FITC group, and the FITC + DINP-200 + HC030031 group to the FITC + DINP-200 group (Fig. 1A,B,C).

### Blockade of TRPA1 alleviated allergic contact dermatitis aggravated by DINP

**Figure 2:**
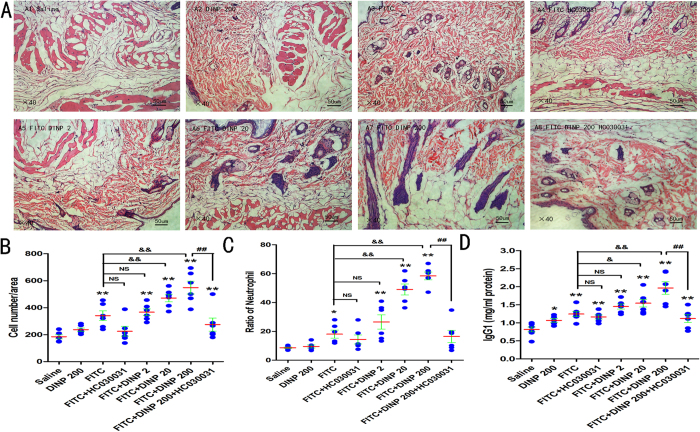
Blockade of TRPA1 alleviated allergic contact dermatitis aggravated by DINP (**A**) The skin from the exposed back of mice, stained with hematoxylin & eosin (H&E). A1, saline group. A2, DINP200 group. A3, FITC group. A4, FITC + HC030031 group. A5-A7, FITC + DINP2, FITC + DINP20, FITC + DINP200 groups. (**B**) Number of inflammatory cells infiltrating the skin. (**C**) Ratio of neutrophils in the peripheral blood. (**D**) Total IgE concentrations (ng/ml). *p < 0.05, **p < 0.01, compared with saline group. NS, no significant difference, ^&^p < 0.05, ^&&^p < 0.01 compared with FITC group. ^##^p < 0.01, FITC + DINP200 group versus FITC + DINP200 + HC030031 group. (n = 7).

To determine the significance of the TRPA1 channel on allergic contact dermatitis, we isolated the back skin of mice and used HE staining to examine pathological reactions after administering HC030031. Histological analysis of the back skins harvested 24 hours after the last challenge revealed that FITC-treated mice showed edema and severe inflammatory cell infiltration in the dermis and subcutaneous tissue (Fig. 2A,B). Compared to the FITC-only exposure mice, these pathological reactions were much more evident in the DINP oral exposure and FITC treatment groups. These aggravated effects were much more obvious with increasing levels of DINP oral exposure. However, when the TRPA1 channel was blocked by administering HC030031, edema and inflammatory cell infiltration in the dermis and subcutaneous tissue were obviously alleviated (Fig. 2A).

Consistent with these effects, the score of inflammatory cell infiltration was sharply decreased when the mice were treated with HC030031, which was shown by comparing the FITC + DINP-200 + HC030031 group with the FITC + DINP-200 group. Compared with the FITC-only exposure group, the score of inflammatory cell infiltration in the FITC + DINP-20 group and the FITC + DINP-200 group increased significantly and very significantly respectively (Fig. 2B).

We further determined the number of neutrophils in the peripheral blood of mice (Fig. 2C). Compared with the vehicle control group, the ratio of neutrophils to white blood cells (WBC) was significantly enhanced in the FITC treatment groups (Fig. 2C). By comparing the DINP + FITC treatment groups with the FITC-stimulation-alone group, we found that the number of neutrophils showed a marked increase with increasing DINP exposure concentrations. More importantly, the number of neutrophils was seen to decrease sharply when the TRPA1 channel was blocked by HC030031 (Fig. 2C).

To validate the alleviating effect of blocking the TRPA1 channel on allergic contact dermatitis, we next investigated the levels of serum IgG1 after HC030031 was administered. Consistent with previously observed pathological changes, FITC stimulated an increase in IgG1 levels, and DINP exacerbated this enhancement and suggested a dose-effect relationship with the levels of IgG1 (Fig. 2D). Notably, blocking the TRPA1 channel inhibited the enhancement of IgG1 induced by DINP, which was shown by comparing the FITC + DINP-200 + HC030031 group with the FITC + DINP-200 group.

These results suggest that the TRPA1 ion channel is closely associated with the development of allergic contact dermatitis induced by DINP, and that this effect could be effectively alleviated by blocking the TRPA1 channel.

### Blockade of TRPA1 inhibited the production of Th2 cytokine IL-13, the levels of IL-6, and mast cell degranulation

**Figure 3:**
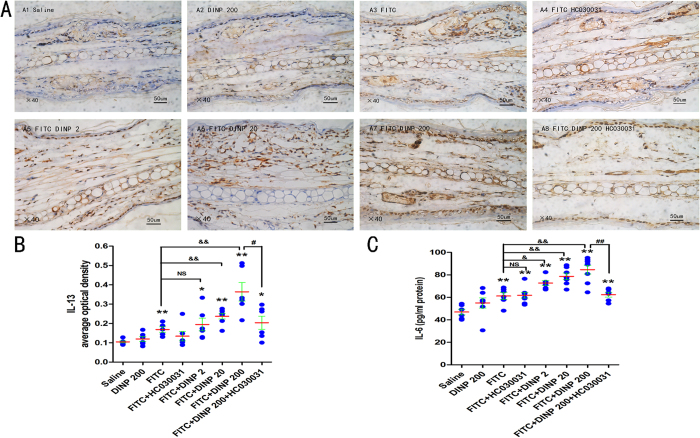
Blockade of TRPA1 inhibited the enhancement of IL-13 and IL-6 induced by DINP. (**A**) Immunohistochemistry for IL-13. (**B**) The expression scores of IL-13. The expression scores were calculated by statistical analysis of optical density of Immunohistochemistry. (**C**) The levels of IL-6. *p < 0.05, **p < 0.01, compared with saline group; ^&^p < 0.05, ^&&^p < 0.01 compared with FITC group. ^##^p < 0.01, FITC + DINP200 group versus FITC + DINP200 + HC030031 group. (n = 7).

Since the Th2 cytokine IL-13 is thought to be a central mediator of the physiological changes induced by allergic inflammation, we investigated the production of IL-13 to further determine the effects of blocking the TRPA1 channel, and the associated mechanisms. A marked increase in the Th2 cytokine IL-13 was seen after exposure to 200 mg/(kg·d) DINP and treatment with FITC (Fig. 3A,B). Interestingly, the exacerbated IL-13 level induced by DINP was almost completely absent when the TRPA1 channel was blocked by HC030031, which was suggested by comparing the FITC + DINP-200 + HC030031 group with the FITC + DINP-200 group (Fig. 3A,B).

Next, we examined the levels of IL-6 in serum since IL-6 is known to promote the production of Th2 cytokines during Th2 differentiation^29^ and an increase in mast cell numbers and reactivity^30^. Similarly, DINP promoted the production of IL-6 in the presence of FITC (Fig. 3C). More importantly, a sharp decrease in IL-6 was seen after administering HC030031 (Fig. 3C).

**Figure 4:**
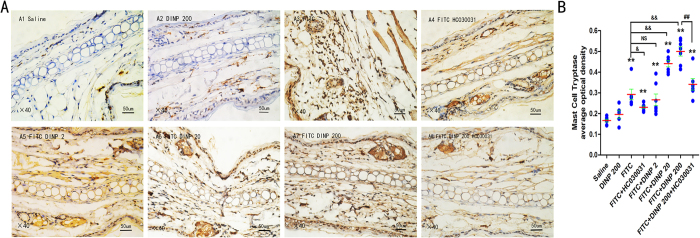
Blockade of TRPA1 inhibited mast cell degranulation aggravated by DINP. (**A**) Immunohistochemistry for Tryptase. A1, saline group. A2, DINP200 group. A3, FITC group. A4, FITC + HC030031 group. A5-A7, FITC + DINP2, FITC + DINP20, FITC + DINP200 groups. (**B**) The expression scores of Tryptase in mast cells. The expression or activation scores were calculated by statistical analysis of optical density of immunohistochemistry. *p < 0.05, **p < 0.01, compared with saline group; ^&^p < 0.05, ^&&^p < 0.01 compared with FITC group. ^##^p < 0.01, FITC + DINP200 group versus FITC + DINP200 + HC030031 group. (n = 7).

We used immunohistochemistry for Tryptase to determine mast cell degranulation (Fig. 4A). In comparison to the mice treated with FITC alone, those treated with FITC and DINP showed an increase in mast cell degranulation as demonstrated by the score obtained when using the immunohistochemistry for Tryptase (Fig. 4B). This effect was inhibited when the TRPA1 channel was blocked by HC030031.

### TRPA1 ion channel is dependent on the activation of NF-κB signaling pathway

**Figure 5:**
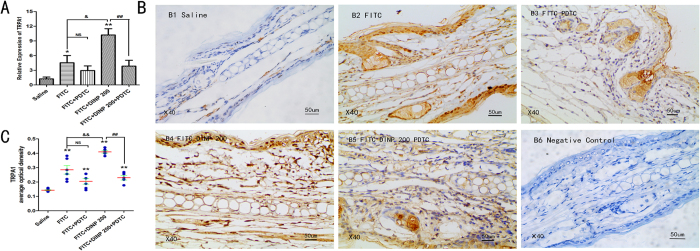
Blocking NF-κB inhibited the expression of TRPA1 (**A**) The effect of PDTC treatment on the expression of TRPA1 mRNA. (**B**) Immunohistochemistry for TRPA1. (**C**) The expression scores of TRPA1. B1, saline group. B2, FITC group. B3, FITC + PDTC group. B4, FITC + DINP200 group. B5, FITC + DINP200 + PDTC group. B6, Negative control. The expression or activation scores were calculated by statistical analysis of optical density of Immunohistochemistry. *p < 0.05, **p < 0.01, compared with saline group; ^&^p < 0.05, ^&&^p < 0.01 compared with FITC group. ^##^p < 0.01, FITC + DINP200 group versus FITC + DINP200 + PDTCgroup. (n = 7).

**Figure 6:**
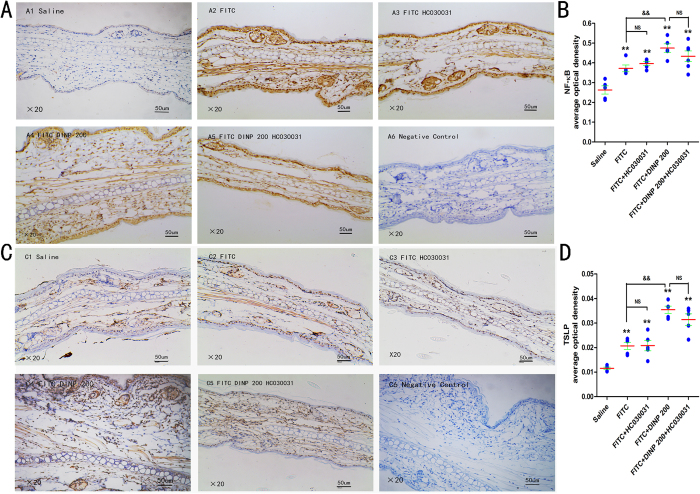
The effect of TRPA1 blockade on NF-κB signaling pathway and the expression of TSLP. (**A**) Immunohistochemistry for NF-κB p65 (phospho S536) (**B**) The activation scores of NF-κB p65 (phospho S536) (**C**) Immunohistochemistry for TSLP. (**D**) The expression scores of TSLP. A1, C1, saline group. A2, C2, FITC group. A3, C3, FITC + PDTC group. A4, C4, FITC + DINP200 group. A5, C5, FITC + DINP200 + PDTC group. A6, C6, Negative control. The expression or activation scores were calculated by statistical analysis of optical density of immunohistochemistry. *p < 0.05, **p < 0.01, compared with saline group; ^&^p < 0.05, ^&&^p < 0.01 compared with FITC group. ^##^p < 0.01, FITC + DINP200 group versus FITC + DINP200 + PDTCgroup. (n = 7).

We next would like to investigate the relationship between TRPA1 ion channel and the NF-κB signaling pathway. Our recent study suggested that DINP exacerbated the activation of NF-κB and that the levels of NF-κB activation were inhibited by treatment with PDTC to block the NF-κB signaling pathway^28^. This study found that the mRNA levels of TRPA1 were also inhibited when PDTC was administered (Fig. 5A), as see when comparing the FITC + DINP-200 + PDTC group and the FITC + DINP-200 group. Immunohistochemical analysis for these same two groups further validated the finding that TRPA1 expression was inhibited when the NF-κB signaling pathway was blocked by PDTC (Fig. 5B,C). However, blocking the TRPA1 channel using HC030031 had no significant influence on NF-κB activation (Fig. 6A,B), as shown by comparing the expression of phospho-p65 from the FITC + DINP-200 + HC030031 group and from the FITC + DINP-200 group (Fig. 6A,B).

Thymic stromal lymphopoietin (TSLP) is thought to be involved in the development of Th2-type allergic disorders, and to be regulated by the NF-κB signaling pathway. TSLP levels in the skin of an ear of exposed animals were investigated. In the presence of FITC sensitization, DINP increased TSLP expression (Fig. 6C,D). It was shown that blocking the TRPA1 channel by HC030031 had no significant effect on TSLP expression, comparison FITC + DINP-200 + HC030031 group to those treated with FITC + DINP-200 group (Fig. 6C,D).

## Discussion

There is controversy regarding the connection between exposure to phthalates and an increased risk of allergic dermatitis^31^. The results from our study suggest that oral exposure to DINP exacerbated allergic dermatitis tissue lesions in FITC-sensitized mice. This aggravation of a CHS response induced by exposure to DINP, was accompanied by an increase in edema and an aggravation of basophil infiltration in the back skin, an enhancement of neutrophils in the peripheral blood, and exacerbating mast cell degranulation. Exposure to DINP also increased production of serum total IgG1 and Th2 cytokine IL-13 and the pleiotropic cytokine IL-6. These findings support the hypothesis that DINP has an adjuvant effect in the CHS response.

TRPA1 reacts with various noxious compounds encountered in our environment or with compounds produced endogenously during tissue injury or drug metabolization^32^. It has been suggested that TRPA1 plays an important role in inflammatory and pruritic responses associated with contact dermatitis^20^. Interestingly, and consistent with an ACD effect, the expression of TRPA1 was seen to be markedly higher in the skin of the ears of FITC-sensitized mice. But more important is the fact that exposure to DINP sharply increased TRPA1 expression. To validate the significance of the TRPA1 channel in allergic contact dermatitis, we used HC030031 to block activation of the TRPA1 ion channel in FITC-sensitized mice exposed to DINP. Our results suggest that injection of HC030031 effectively prevented the development of ACD in mice that were orally exposed to DINP and/or FITC-sensitized. HC030031 antagonism can be clearly identified through a number of changes, such as the downregulation of IgG1 levels, ameliorating inflammatory cell infiltration, reduction of mast cell degranulation and a decrease in IL-6 and IL-13 to varying degrees. Similarly, the edema of back skin was also attenuated. This strongly indicated that TRPA1 ion channel antagonists successfully alleviated the effects of inflammation and supports the hypothesis that activation of the TRPA1 ion channel is involved in mediating the adjuvant effects induced by DINP.

IL-6 receptor variant has been reported as a risk factor for atopic dermatitis^33^. IL-6 levels were found to be elevated in asthmatic patients^29^. It is known to regulate the fate of CD4 T cells by promoting the Th2 cytokine and inhibiting Th1 differentiation^29^. IL-13 is a typical Th2 cytokine and is thought to be a central mediator in the physiological changes induced by allergic inflammation. This study demonstrated that DINP induced the enhancement of IL-6 levels and the production of the Th2 cytokine IL-13, and that blocking the TRPA1 ion channel led to a decrease in IL-6 and IL-13 levels.

IL-6 was reported to promote mast cell proliferation, maturation, and reactivity^30^. In this study, we have shown that mast cell degranulation was aggravated by exposure to DINP and FITC, and ameliorated by blocking the TRPA1 channel. This effect might be due to changes in the important regulator IL-6.

TSLP is required for the development of Th2-type contact hypersensitivity induced by FITC in combination with dibutyl phthalate^7^. This cytokine is a critical factor linking responses at interfaces between the body and the environment to Th2 responses. TSLP has been reported to be regulated by transcription factor NF-κB in humans and mice^34^. Our recent study also suggested that DINP exacerbated expression of TSLP through NF-κB^28^. However, the relationship between TRPA1 activation and the NF-κB signaling pathway is not clear. In this work, we investigated this relationship by blocking activation of the TRPA1 channel and the NF-κB signaling pathway, by injecting HC030031 and PDTC, respectively. Results showed that blocking NF-κB by PDTC inhibited TRPA1 expression, while blocking the TRPA1 channel by HC030031 had no significant effect on the activation of NF-κB, nor on the expression of TSLP. These findings suggest that activation of TRPA1 depends on the activation of the NF-κB signaling pathway and enhancement of TSLP expression.

Therefore, we propose a mechanism to explain the aggravation caused by DINP exposure on the development of allergic dermatitis in mice. In the presence of FITC, exposure to DINP enhances TRPA1 expression and/or activation of TRPA1 by activation of the NF-κB signaling pathway and increased production of TSLP. This activation or enhancement of gene expression increases secretion of IL-6 and Th2 cytokines, and which in turn promotes the development of allergic dermatitis in mice.

This study suggests that blockade of TRPA1 might ameliorate the aggravation effect on ACD induced by DINP. It demonstrated that activation of TRPA1 depends on the activation of the NF-κB signaling pathway and enhanced TSLP expression. This provides new insight onto the mechanism behind the adjuvant effect induced by DINP exposure on the development of ACD and will therefore help in understanding how to control allergic dermatitis.

## Methods

### Chemicals

DINP (>99%), DBP (>99%), FITC, pentobarbital sodium and formalin solution (4%) were bought from Sigma-Aldrich (St. Louis, MO, USA). Tween-20 and Tween-80 were purchased from Amresco (Solon, OH, USA). HC030031 was custom synthesized by Cayman Chemical Company (Ann Arbor, Michigan, USA). ELISA kits (mouse) for total IgG1, IL-6 were obtained from Blue Gene (Shanghai, China) and eBioscience (San Diego, CA, USA), respectively. The protein test kits were provided by Beijing Dingguo Changsheng Biotechnology Co. LTD (Beijing, China).

### Construction of ACD model of mice

Male Balb/c mice were purchased from Wuhan Institute of Biological Products Co., Ltd., approximately 8–9 weeks old, weighing between 20–22 g. Mice were housed under SPF raising conditions. The testing procedures were approved by the Office of Scientific Research Management, Central China Normal University on 20 December, 2015 (approval ID: CCNU-IACUC-2016-003). All methods were performed in accordance with the approved guidelines and regulations. The protocol for exposure and sensitization was as follows. Balb/c mice were gavaged with saline or DINP (2, 20 and 200 mg/(kg·d)) from day 1 to 21. On days 22 and 23, 120 ul of saline or 0.5% FITC (in 1:1 acetone/DBP) was smeared on to the shaved backs to sensitize mice. The mice were challenged with 20 ul of saline or 0.5% FITC to the right ear on the 28^th^ day.

### NF-κB blocking and TRPA1 blocking

Mice were injected with 60 mg/kg bw/day pyrollidine dithiocarbamate (PDTC) to block NF-κB 1 h prior to sensitization or challenge on days 22, 23 and 28. HC-030031 (50 mM HC-030031 stock in DMSO and 0.2% Tween 80 in saline) was used as a selective TRPA1 antagonist. On days 22, 23 and 28, the TRPA1 antagonist HC-030031 was injected (50 mg/kg, i.p.) 1 h prior to challenge. In addition, after the FITC provocation on the 28th day, HC-030031 (50 mg/kg, i.p.) was injected twice at 8 h and again at 16 h.

### Determination of IgG1

Within 24 hours after the end of the exposure experiment, serum samples were taken from heart blood and stored at −80 °C prior to analysis. The total IgG1 levels of these samples were measured using an ELISA kit from Blue Gene (Shanghai, China) according to the manufacturer’s instructions.

### Cell counts for the peripheral blood

Peripheral blood was collected from the tails of the mice. The number of white blood cell and neutrophils were recorded using a haematology analyser (MTN-23, China).

### ELISA analysis of chemokine release in the skin supernatants

In brief, frozen skin or right ear tissues were placed in liquid nitrogen, then pulverized with a chilled mortar and pestle into powder. 10% PBS was then added to the powder. The supernatant was collected at 24 h, centrifuged at 10000 rpm for 15 min at 4 °C, and stored at −80 °C prior to ELISA analysis. IL-6 was quantified by ELISA analysis according to normal laboratory protocols. The sensitivity limit for IL-6 was 15 pg/ml.

### Real-time polymerase chain reaction (PCR) and RT-PCR analysis

Total cellular RNA was extracted using the TRIzol method. cDNA synthesis was performed using a high-capacity RNA-to-cDNA Kit (Takara Biotechnology Dalian, China). For real-time quantitative PCR (qPCR), each sample was run in triplicate and normalized to housekeeping gene GAPDH expression. Ct values were determined using Light-Cycler 480 software and averaged. Relative quantification was determined by the ∆∆Ct method. The forward and reverse-specific primer sequences, the size of the amplified fragment and the annealing temperature for TRPA1 were 5′-TCTCCACCTGGCAGCAAAAA-3′ and 5′-CATGGAGGCGTGATGCAAAG-3′, 103 bp, 54–57 °C; and for GAPDH were 5′-AGTGCCAGCCTCGTCCCGTA-3′ and 5′-CAGGCGCCCAATACGGCCAA-3′, 137 bp, 54–57 °C.

### Histological and immunohistochemical analysis

Circular 8-mm punch biopsies were excised from ears and nape and fixed in 4% formaldehyde and embedded in paraffin. Sections were cut at 3 μm, mounted onto slides, and stained with H&E according to standard procedures. After dewaxing, rehydration and antigen retrieval, the sections were incubated with 0.3% hydrogen peroxide and blocked by appropriate normal serum. Sections were subsequently incubated overnight at 4 °C with monoclonal antibodies: anti-phospho-p65 (s536) (1:200, Abcam, MA, USA), anti-TRPA1 (1:50, Abcam, MA, USA), anti-Mast Cell Tryptase (1:50, Abcam, MA, USA). Antibody binding was then detected by biotinylated immunoglobulins and avidin-biotin peroxidase complex. The reaction product was visualized by DAB complexes. The negative control was obtained by omitting the primary antibody. Sections were washed again, counterstained with hematoxylin, dehydrated, cleared, and mounted in DPX (Sigma-Aldrich).

## Additional Information

**How to cite this article**: Kang, J. *et al*. TRPA1 mediated aggravation of allergic contact dermatitis induced by DINP and regulated by NF-κB activation. *Sci. Rep.* **7**, 43586; doi: 10.1038/srep43586 (2017).

**Publisher's note:** Springer Nature remains neutral with regard to jurisdictional claims in published maps and institutional affiliations.
